# Defining the Role of the Online Therapeutic Facilitator: Principles and Guidelines Developed for Couplelinks, an Online Support Program for Couples Affected by Breast Cancer

**DOI:** 10.2196/cancer.3887

**Published:** 2015-04-14

**Authors:** Wendy Carter, Karen Fergus, Saunia Ahmad, Deborah McLeod, Joanne Stephen

**Affiliations:** ^1^Private PracticeToronto, ONCanada; ^2^Department of PsychologyYork UniversityToronto, ONCanada; ^3^Patient and Family Support ServicesOdette Cancer CentreSunnybrook Health Sciences CentreToronto, ONCanada; ^4^Psychosocial Oncology TeamCapital District Cancer Care ProgramQueen Elizabeth II Health Sciences CentreHalifax, NSCanada; ^5^Psychosocial OncologyDepartment of Oncology, Faculty of MedicineUniversity of CalgaryCalgary, ABCanada

**Keywords:** couples, cancer, Internet, online therapist, psychosocial support, therapeutic alliance

## Abstract

Development of psychological interventions delivered via the Internet is a rapidly growing field with the potential to make vital services more accessible. However, there is a corresponding need for careful examination of factors that contribute to effectiveness of Internet-delivered interventions, especially given the observed high dropout rates relative to traditional in-person (IP) interventions. Research has found that the involvement of an online therapist in a Web-based intervention reduces treatment dropout. However, the role of such online therapists is seldom well articulated and varies considerably across programs making it difficult to discern processes that are important for online therapist involvement.In this paper, we introduce the concept of “therapeutic facilitation” to describe the role of the online therapist that was developed and further refined in the context of a Web-based, asynchronous psychosocial intervention for couples affected by breast cancer called Couplelinks. Couplelinks is structured into 6 dyadic learning modules designed to be completed on a weekly basis in consultation with a facilitator through regular, asynchronous, online text-based communication.Principles of therapeutic facilitation derived from a combination of theory underlying the intervention and pilot-testing of the first iteration of the program are described. Case examples to illustrate these principles as well as commonly encountered challenges to online facilitation are presented. Guidelines and principles for therapeutic facilitation hold relevance for professionally delivered online programs more broadly, beyond interventions for couples and cancer.

## Introduction

There has been a steady increase in the number of Internet-delivered psychological interventions addressing a range of different issues that include chronic medical conditions (see [1,2]), eating disorders [3-5], substance use (see [6] for review), depression and anxiety [7-9], and cancer (eg, [10,11]). Although research suggests that such online programs are quite effective (eg, [12]), few studies have examined the components of an online intervention that contribute to program adherence and positive outcomes. Such lack of knowledge is problematic given that online programs demonstrate higher attrition rates relative to traditional in-person (IP) therapy, rendering the significant outcomes achieved applicable to only a subsample of participants [13,14]. Specifically, self-help Web programs that are entirely self-guided (ie, have no contact with or guidance from an online expert or professional incorporated into the program) demonstrate much higher attrition rates with program completion ranging anywhere from 1% [15,12] to 53% [16]. There is compelling evidence that involvement of an online clinician is associated with better outcomes for Internet-delivered interventions (see [9,17] for reviews). A recent qualitative study with individuals that participated in online interventions without an online therapist found that the most commonly cited reasons for program dissatisfaction and disengagement included the lack of personalized support, feedback, and guidance [18,19].

Despite its potentially critical role in program adherence and outcome, there has been minimal discussion and guidelines in the literature on how best to provide therapeutic support within the context of an online, primarily self-guided, psychosocial intervention. Online interventions are different from IP therapy in that much of the intervention is delivered by way of the Web program and an individual’s interaction with the program (eg, reviewing content, logging behavior). The online clinician, when included, may have differing tasks depending on the program, but generally is there to provide support with the objective of ensuring compliance with the intervention. In contrast, the IP therapist provides the intervention itself. Therefore, the 2 types of intervention differ in their format and hence the role of the clinician. Still, as trained clinicians, online therapists/facilitators are likely to draw on their general psychotherapy skills, such as empathy and validation in the provision of support. Compared to IP therapists, online clinicians are not privy to the nonverbal communication and paralinguistic cues of their clients [20]. Not surprisingly, relative to IP clinicians, online clinicians express a lack of clarity around how to fulfill similar objectives as the IP therapist using text-based formats for interaction [21].

This paper takes a step toward addressing this gap by defining the role of the online clinician, referred to in this paper as the online facilitator, and by presenting principles and guidelines for online therapeutic facilitation that were developed and refined in the context of a novel Web-based intervention for couples coping with breast cancer. The program, called Couplelinks, was previously developed and tested in a Phase I pilot trial [22] and is presently being evaluated in a multisite randomized controlled trial (RCT) [23]. The observations of the facilitation process in the pilot trial led to greater elaboration and precision of the facilitation guidelines and were formally described in the Couplelinks Program Facilitation Manual [24] for the RCT. The principles and strategies discussed are based on what was learned from the pilot trial.

Interaction with the online facilitator in this program is primarily via text-based asynchronous online communication. The online facilitator role was developed with the vision that it need not entirely mirror the IP therapist role given the obvious differences between a self-guided Web-based intervention program and IP therapy, but instead be defined differently bearing in mind the potential for unique advantages specific to the technological medium.

In this paper, we review the modest literature on the various ways professionally offered support in online interventions has been defined and provided. This review is followed by a proposed definition of the online facilitator and guiding principles for online facilitation in the context of Couplelinks. Challenges for couple participation and engagement using the online modality are discussed and highlighted with use of case examples from the Couplelinks pilot study.

## Research on the Utility of an Online Clinician for Web-Based Interventions

Inclusion of a professionally trained support person in a Web-based intervention varies considerably across programs in terms of the role and the nature of his or her interaction with participants. Methods of online support that have been used and found to increase program adherence include online, telephone, or mailed reminders and brief written descriptions on how to use the program [25-27]; online feedback aimed to emphasize new learning and encourage ongoing engagement [28,29]; and/or telephone contact [7,30]. With respect to program effectiveness, 2 meta-analyses of studies comparing Internet-based interventions for depression and anxiety disorders to a no-treatment control group found that Web-based interventions with therapist support revealed larger effect sizes than Web-based interventions without therapist support [7,9]. Palmqvist et al’s [17] quantitative synthesis of several Internet-based interventions found that the amount of online therapist support provided in an intervention was positively correlated with outcome, such that more support related to greater benefits derived from the program.

Few studies have compared the same online intervention with and without therapist guidance and for the few that have been done, the results are equivocal with some finding superior outcomes for an online therapist-guided program over a non-therapist-guided version [31,32] and others finding no significant difference [33,34]. However, the design of the programs themselves, the populations served, and the function and nature of therapist involvement in these programs varied considerably making comparisons difficult. For a number of online self-help programs, “support” was operationalized as automated reminder messages or program instruction (eg, [25,27,28,35]). In other studies, the therapist provided more tailored “feedback” to clients; for instance, specific strategies or access to additional relevant resources [36] or a discussion regarding the interpretation of assessment outcomes and the client’s reaction to this information [37]. Berger and colleagues [34] found no difference between therapist-supported and unsupported versions of an online depression intervention. However, therapist support in their study involved weekly generic email responses meant to motivate participants but not necessarily provide any tailored feedback on process or progress. In contrast, Lancee and colleagues’ [31] online intervention for insomnia found significantly greater outcomes for the therapist-guided group compared to the unsupported group. Participants in their therapist-supported group received emails that included tailored feedback on their progress and support and encouragement to complete modules, including suggestions on how to continue with the program. Therapists had the ability to log in and review participants’ progress on assigned exercises and integrate participants’ own words into the therapist’s feedback. In contrast, the unsupported group received prescripted automated email reminders to complete the program and had someone available to contact for questions regarding procedural or technical issues. Taken together, the research suggests that therapist involvement entailing a more individualized approach to online facilitation rather than mere generic responses is critical to outcomes.

Todkill and Powell [19] assessed experience of participants in a RCT of a self-help cognitive behavioral therapy program that included automated email reminders to log in and complete the program. Participants reported that the absence of a message tailored specifically to them as individuals was a major drawback of the program. Similarly, Mathieu et al [18] found that although most participants of an Internet-based psychological program liked if not preferred Internet-based delivery of an intervention because it was flexible, easy to use, and not burdensome, they also reported feeling restricted, disconnected, and unmotivated to continue because of the lack of an online support person to provide personalized feedback and understanding, and the lack of someone who was available to respond to questions and take into consideration their individual circumstances. Finally, Berger and colleagues [34] found that 73% of participants in a non-therapist-supported group desired contact with an online therapist.

## Reconceptualizing the Therapist-Client Relationship in Web-Based Interventions

The association between therapeutic alliance and outcomes in IP settings is well established [38,39]. When comparing working alliance client ratings online versus IP therapies, no significant difference in ratings were found, suggesting that a strong therapeutic alliance can be established equally in IP and online mediums (see [40] for review). Different from IP therapy, however, alliance ratings for online interventions have reportedly had a small to no significant effect on outcome despite the high level of therapeutic alliance achieved in them [41-43].

How can we reconcile the findings that the therapeutic alliance is a major factor that explains outcomes of IP therapy but not Web-based interventions, yet both forms of intervention demonstrate comparable efficacy? One possible explanation for this seeming paradox is that the alliance with an online therapist is qualitatively different from that of the alliance formed with an IP therapist because it emerges from the synergistic effect of the website components and the online clinicians’ responses [44]. Peck [45] addresses this paradox by reinterpreting the well-established finding that the therapy relationship contributes to outcomes by suggesting that it is not the relationship itself but the processes it activates within a client that impacts outcomes. As he writes, “In contemplating this conundrum, it may be advantageous to construe the therapeutic relationship not as one of the common factors, but as the vehicle or channel that facilitates (or hinders) the activation of the remaining ‘true’ common (and specific) factors” [45]. Peck [45] further elaborates by suggesting that in IP therapy, the therapist is typically the only source of delivering the common and specific therapeutic factors, thus components of interpersonal skill in-person will largely determine how well and if therapeutic change processes will be activated. On the other hand, in Web-based interventions, various evidence-based components of therapy are delivered in a structured way via the website itself. Cavanagh and Millings [44] suggest that the significance of other factors, such as support and accountability to the program, will be more salient for the online therapist relationship.

In what ways does the online therapist provide this support and encourage such engagement? Despite recommendations that online interventions require the development of specific skills [46], little work has been done to date to articulate the type of specialized skills required to provide the most effective online facilitation. The way in which the therapist provides “support,” “guidance,” “assistance,” or “feedback” is not well defined for the majority of Web-based interventions [17]. Furthermore, the amount of time and level of engagement of the online therapist is not consistently defined, measured, and reported alongside empirical findings.

Mohr et al [47] propose that the online therapist’s role is that of “supportive accountability” and interventions are meant to support progress through and completion of the program using various technological components such as email or phone calls. They further elaborate on the role by suggesting that the online coach is seen as a trustworthy and benevolent person with expertise in the intervention and demonstrates presence and accountability to the program objectives through their interventions. Part of supporting accountability includes clarifying expectations regarding how various aspects of the intervention relate to the benefits clients would experience, making the intervention meaningful and hence increasing compliance. Similarly, Warmerdam and colleagues [29] suggest that the primary role of the online facilitator is to offer feedback with the aim of helping the client work through the program rather than to provide advice or foster a therapeutic relationship per se. In their study, the feedback content consisted of “showing empathy by letting participants know that the coach had read the assignments, being positive by giving compliments on what the participant had done, and giving suggestions on how to continue with the course” [29].

Despite obvious limitations in the level of interaction that can be achieved with clients via a Web-based program, there are several advantages. For example, the clinician can review participant’s progress via the data entered online and provide feedback accordingly [48]. Furthermore, Stephen and colleagues [49] reported that facilitators found the physical distance of the online format helped them to better manage the emotional content of the group. Online facilitators in their study found that the act of writing increased their own self-awareness and mindfulness of the specific clinical skills and interventions being incorporated.

## Couplelinks: Program Rationale and Description

Providing psychosocial support via the Internet for people dealing with serious chronic illness such as cancer is particularly compelling when considering the potential geographical, physical, or psychological barriers that may make IP therapy impossible. Serious illnesses such as cancer affect not just the person with the illness, but their family members and their intimate partner [50]. Cancer is destabilizing to the relationship system and invariably results in significant relationship reorganization and strain [51]. The rather modest collection of research examining online facilitation with cancer populations focuses predominantly on Internet-based support groups for patients (eg, [52-60]). Online interventions for couples dealing with cancer, however, are notably absent. This oversight is problematic given that cancer has a profound impact on both the individual and the relationship system.

A well-established finding is that younger, mainly premenopausal breast cancer survivors (age 50 or younger) and their partners are significantly more likely than older couples to experience relationship distress and poorer quality of life [61,62], and are more likely to continue experiencing declines in relationship functioning and quality of life 5 and 10 years after treatment completion [63,64]. Younger couples dealing with cancer experience multiple barriers to traditional counseling that limit the likelihood that these couples will seek support. Factors such as being in active cancer treatment may result in reduced inclination on the part of individuals to seek out couples counseling due to additional appointments, particularly if they are coping with the effects of treatment [65]. As well, younger couples tend to lead busier lives, as they juggle work and family commitments, and may have difficulty scheduling and obtaining professional support, particularly if they have young children [62,65-67]. Moreover, if offered in hospital settings, psychosocial support is likely limited to daily working hours, which may prove a challenge for caregiving partners based on their employment.

Such barriers were taken into consideration in the development of Couplelinks—a novel, professionally facilitated, asynchronous online intervention designed to enhance relationship adjustment and dyadic coping, and reduce individual distress of young couples affected by breast cancer. The program is based on the premise that partners in intimate relationships vary in the degree to which they feel identified with the relationship, also referred to as “couple identity” or “we-ness.” When partners experience their relationship as part of their sense of self, they are more likely to think about issues and events from each other’s perspective and view stressors as shared. Consequently, such partners engage in greater perspective-taking, empathy, and interpersonal support and therefore experience greater relationship satisfaction [68,69]. Greater levels of we-ness better equip couples to cope with various stressors related to breast cancer and therefore experience lower levels of individual and relational distress [51,70]. Couples who construe the cancer as a shared problem are better able to engage in mutually supportive interactions that promote adjustment. Indeed, research has found that couples with higher levels of we-ness or dyadic coping in relation to breast cancer experience better adjustment (eg, [70-73]).

Couplelinks focuses on enhancing such we-ness through the use of experiential exercises designed to improve couples’ communication, perspective-taking ability, and mutual understanding and empathy in relation to breast cancer. The program is structured into 6 dyadic learning modules designed to be completed on a weekly basis in consultation with a Couplelinks facilitator through regular, asynchronous, online text-based communication at the end of every module. The Couplelinks facilitator, who is a mental health professional with experience in oncology, guides the couple throughout the program. Each weekly module assumes the following basic structure that the partners engage in on their own in the following order: (1) a theoretical component that explains a key relationship principle, (2) a dyadic, experiential exercise intended to assist the couple in grasping the principle, and (3) a feedback component that each partner is asked to complete on his or her own. The facilitator then reviews the modules and logs text-based feedback via the website. In addition to such online, asynchronous, text-based contact, the facilitator schedules phone check-ins with the couple after completion of Modules 2 and 4 to discuss any issues with the program and reinforce motivation and engagement with the program. The facilitator is also available as needed.

The weekly modules are broken down into steps, some of which are completed separately by each partner and some that are completed jointly as a couple. Typically, a module starts with activities that partners complete separately, followed by a dyadic activity that incites discussion and new learning for the couple. Such learning is consolidated in the last stage of the module when partners separately answer a series of questions assessing what the partners learned and what benefits they gained from completing the module, if any. Once both partners complete a module, an email notification is automatically generated and sent to their online facilitator who then logs in to a back-end administrative interface to review the couple’s entries to the dyadic learning module. The online facilitator provides tailored feedback on the couple’s responses to the module via the Dialogue Room, which is a 3-way virtual bulletin board embedded within the website. The partners receive an automatic email alert indicating that their online facilitator has provided them with feedback in the Dialogue Room and partners can only review the feedback by logging in to the secure Couplelinks website. The facilitator ends their feedback response with a description and explanation of the learning objectives of the upcoming module and a due date for its completion, typically a week from the date the feedback was sent. Although feedback is tailored to the couple and what they logged in their modules, a standard script of the description of the subsequent module is available to the facilitators in the facilitation manual [24] that can be tweaked to blend with the content of their feedback.

The Dialogue Room acts as a forum where participants and the facilitator can communicate with each other. For instance, the couple can raise concerns and ask questions as well as let their facilitator know if they need more time or had something unexpected happen that will delay their progress. The facilitator can also use the Dialogue Room to check in with a couple if he/she has not heard from them and they are behind schedule. Facilitators log any interactions that occur with the couple outside the Dialogue Room in a section viewable in the administrative interface called “Contact Notes.” This section includes a summary of scheduled and unscheduled phone calls and emails. The module logs, Dialogue Room, and Contact Notes provide the basis with which to assess whether facilitators are adherent with the principles of facilitation as outlined in the treatment manual.

## Online Therapeutic Facilitation of Couples

The Couplelinks facilitator’s role is to provide encouragement, safety, and a sense of structure through regular online communication with the couple. We conceived of the role of the Couplelinks facilitator as that of an expert guide who functions to support and encourage the couple’s learning process and enhances adherence to the program. The Couplelinks facilitator is a trained mental health professional with expertise in psychooncology and couple interventions.

Although not engaging in psychotherapy, the facilitator draws on his/her clinical skills and judgment when crafting customized feedback to the couple on completion of a dyadic learning module and as problems and unexpected situations occur, such as when the couple does not complete a dyadic learning module by the expected time or if one partner is less responsive than the other. We termed this style of facilitator-couple asynchronous online interaction as “therapeutic facilitation.” The term “facilitation” connotes providing assistance to move an action or process forward with greater ease. We see this term as accurately capturing the online facilitator’s role in the context of a primarily self-guided Web-based intervention, which includes assisting couples as they progress through the intervention by clarifying the objectives of each dyadic learning module, answering questions, providing psychoeducation, maintaining structure, encouraging commitment in order to maintain momentum, and validating and reinforcing the learning and insights derived by the couples from the exercises.

The underlying theoretically informed objective of Couplelinks, as described previously, is to enhance couple’s we-ness so that the couple perceives and approaches the cancer as a shared problem. Thus, the overarching goal for the online facilitator when formulating responses to validate couples’ insights and reinforce gains—whether textual or by phone—is to enhance their sense of the illness being a shared experience and accentuate their shared strengths and experiences around this stressor.

The reasoning for employing therapeutic facilitation in the Couplelinks program was based not only on the need to maintain adherence, but on the premise that couples experience the most benefit when *both* partners remain equally engaged and motivated in the program, are able to easily understand how to navigate the program, and feel they are on the right track in terms of their progress. Complex processes such as these cannot be programmed into a computer but require a skilled person on the other end. Therefore, online facilitators play a necessary supporting role in structuring the exercises by drawing on their therapeutic skills to encourage the couple’s open discussion and commitment to the intervention and their shared progression through the program.

Given that the online facilitator is supporting the couple in proceeding through and benefiting from the exercises rather than providing couple therapy, the online facilitator does not engage in certain clinical techniques as an IP therapist would, such as directly challenging partners’ unconstructive behaviors or suggesting alternative interpretations of each other’s behaviors. Instead, emphasis is on skills such as highlighting what the couple shares, vis-à-vis the couple’s responses to the modules. For instance, the online facilitator encourages equal participation of both partners by consistently incorporating comments made by both couple members in their Dialogue Room feedback responses. Additionally, if partners provide differing opinions for dealing with a problem within their relationship and explicitly note their frustrations, like the IP therapist, the online facilitator might note the way in which partners approach problems differently by providing a response in their textual feedback, such as: “It sounds like, even though you may approach things differently, both of you are dedicated to figuring this out and resolving this issue that is causing both of you stress.” The online facilitator, however, does not engage in in-depth exploration of emotions, but frames feedback in such a way that highlights the couple’s strengths and insights gleaned from completing the exercises.

## Facilitator Principles

To guide and standardize the delivery of online facilitation for the Couplelinks RCT, guiding principles and specific strategies were developed. The principles are not mutually exclusive; the facilitator often employs several of these strategies in a single response to a couple. All interactions described subsequently are asynchronous and occur via the Dialogue Room unless indicated otherwise (eg, phone call or emails). Research Ethics Boards of the institutions where the participants were being recruited approved of the Phase I and later Phase III trials. All participants enrolled in the trials were informed of the study objectives, risks, and consequences and consented to participate.

### Collaboratively Developing a Timeline

During the introductory phone call, the online facilitator educates the couple on time commitments and the need to maintain a relatively weekly schedule in completing the exercises, encourages partners to come up with a tentative timeline for completing the dyadic learning modules, and to identify times during the week when they would complete the shared components of the dyadic learning modules. Although the program requires no more than 1 hour commitment per week, the added burdens associated with cancer can make even minimal obligations stressful and thus lessen the potential benefits of the program. The online facilitator encourages an open 3-way discussion that helps the couple examine all their current and upcoming obligations, and allows the facilitator to consider along with the couple how to work around any obstacles to their participation in the program, thus maximizing the likelihood that they will complete the program and obtain the greatest degree of benefit.

### Encourage Open Dialog Not Avoidance

Clearly communicating the online facilitator’s willingness to hear the partner’s feedback to the program, regardless of whether it is positive or negative, is key to establishing and maintaining open communication. The online facilitator cultivates an atmosphere of openness and curiosity about all aspects of the couple’s experience. This means that online facilitators acknowledge and directly address couples’ negative comments regarding the program. Importantly, this behavior also serves to model the concept of open dialog within the couple relationship. As well, although contact with the couple primarily occurs online via text, in order to maintain the couple’s momentum and commitment to the program, we have found it essential that the online facilitator also use brief telephone consultations with couples as necessary. For instance, when there have been significant lapses in online communication, the online facilitator may call the couple to inquire as to what happened.

### Create a Virtual Therapeutic Space

The online facilitator takes advantage of the convenience and accessibility of the online environment to create a safe, supportive therapeutic space in ways that are likely not possible in traditional psychotherapy, such as by responding promptly via the Dialogue Room to questions posted by couples. In contrast to IP therapists that are typically available on a weekly basis at a set time, online facilitators have the opportunity to be more accessible to couples throughout the week. Couplelinks online facilitators are expected to respond to couples’ messages within 24 hours in order to demonstrate their commitment to couples’ timely progress through the program as well as to model frequent engagement. The online facilitator communicates availability, presence, and commitment by responding quickly via text in the Dialogue Room as well as with phone calls, the latter being used if the couple is not responding via the Dialogue Room. Additionally, online facilitators are expected to log in to the administrative interface to review each couple’s progress through the substeps of the module and whether it appears they will complete by the agreed-upon deadline, otherwise providing gentle reminders to encourage them to complete by the due date they agreed to in collaboration with their facilitator.

A common situation in which online facilitators’ online communication serves the dual role of demonstrating their commitment to couples while also modeling genuineness and empathic caring occurs when couples fall silent. Online facilitators are expected to consistently check in with couples when there are unexpected lags between starting and completing an exercise, but do so in ways that are meant to be supportive and encourage accountability to the program. Such a situation provides the facilitator with the opportunity to openly acknowledge and express concern regarding the couple’s silence, which often serves to strengthen the couple-facilitator relationship, reduce isolation, and foster program compliance. This also represents a critical moment when the facilitator can explore the barriers with a couple and problem-solve with the couple about ways to proceed.

### Encourage Structured Flexibility

The online program allows the couple to participate in the privacy of their own home and set their own pace. Although the flexibility of an online program is an unquestionable strength, a lack of structure can also be a drawback in that partners may take the program for granted and easily delay completing the module. In this regard, the online facilitator’s task is to strike the right balance between acknowledging the need for flexibility and setting agreed-upon deadlines. Couplelinks online facilitators are asked to employ a “friendly but firm” stance with couples right from the beginning of the program. For example, when online facilitators are orienting new couples to Couplelinks, they emphasize upfront how couples need to set aside time on a weekly basis to work through each module and encourage the couple’s involvement in problem solving to carve out the time necessary to complete the exercises on a weekly basis. Including the couple in this discussion is meant to strengthen the couple’s commitment and accountability to complete the program. Online facilitators also provide suggestions and help to problem-solve when obstacles arise (as they often do while undergoing or recovering from treatment) and solicit couple involvement in setting revised deadlines when existing ones are missed.

### Engage Both Members of the Couple

In general, equally engaging both members of a couple can be difficult. When one member has initiated the process, the other member may view him- or herself on the periphery of the experience. This is a considerable risk in the context of breast cancer where many male partners, although often eager and willing to help their partners, may view themselves as sitting on the sidelines. In this sense, the online facilitator’s objective during the introductory telephone call before they commence the program is to acknowledge each partner’s individual experience of the disease and personal motivation for participation, highlight the impact of the disease on the couple, and articulate the invaluable and active role that a partner may play in a woman’s recovery. On a weekly basis, a key aspect of the online facilitator’s comments to the couple is acknowledgment and integration of *both* members’ experiences as expressed in the feedback component for each module, which serves to reinforce the involvement and importance of both partners through the program.

In situations where one member of the couple initiates an email outside the Dialogue Room to the online facilitator without including the other partner, the online facilitator will include the absent partner in the reply to maintain the “3-way conversation” format. This is meant to minimize the formation of alliances with the online facilitator that excludes the other partner. Whenever possible, however, the online facilitator will utilize the Dialogue Room to respond to partner inquiries rather than resort to email communication because of the Dialogue Room’s security features and because it automatically engenders the 3-way conversation format as it is always (and only) accessible to both members of the couple.

### Reinforce New Learning

The online facilitator reviews module content and feedback and more clearly articulates and emphasizes emerging insights and positive experiences for the couple. This may involve the online facilitator accentuating insights that partners share about themselves, each other, their relationship, or a given module. This may also involve providing psychoeducation about the module as it pertains to the couples’ reflections. Couples vary in terms of their strengths and areas of challenge. Therefore, some couples may indicate that they did not derive a shift in perspective as the module addressed an already established practice or strength in their relationship. In this case, the online facilitator demonstrates their attentiveness to what the couple is expressing by acknowledging and validating an existing relationship strength.

### Manage Emotional Content

A diagnosis of breast cancer signifies a crisis in the life of a couple. The online facilitator must attend to, manage, and, if need be, contain the emotional content that emerges throughout the course of the program. This is done by constructing Dialogue Room feedback that validates and normalizes the range of emotional responses of couples to different aspects of the program and responding to strong reactions voiced by both or either partner in a supportive way. Where the emotional content is indicative of acute distress, the online facilitator highlights his or her availability to the couple through the Dialogue Room or by phone.

## The Application of Facilitator Principles to Common Challenges

This section describes 4 common challenges in relation to online couple facilitation that came to light during the pilot study. The examples presented subsequently illustrate the ways in which the online facilitator used specific strategies related to the principles of facilitation described to increase the couples’ engagement and address common challenges. The examples of online facilitator responses to couples are taken from the Couplelinks Program Facilitation Manual [24].

### Challenge #1: Differential Involvement of Partners

As described previously, one challenge to consistent participation and the desired outcome of a strong bond within a couple is ensuring equal engagement by both members of the couple in the program. Partners vary in terms of their levels of awareness of the impact of cancer on their relationship as well as their interest and motivation to take part in the program. For couples dealing with breast cancer, it was fairly typical for a male spouse to indicate that even though he agreed to participate in the program, his decision to participate was largely motivated by a desire to support his partner (rather than help himself too). Although differential involvement of partners may also pose a challenge for “offline” counseling, the IP therapist has the added benefit of being able to physically observe the couple dynamic and has greater opportunity to directly address any discrepancy in the moment, which may not be as apparent to the online facilitator who is working via asynchronous communication. Therefore, it was critical that the online facilitator connect with each member of the couple in “real time” over the phone, prior to program commencement, and use this time as an opportunity to highlight the way in which cancer creates havoc in both individuals’ lives and profoundly impacts them as a couple, not just as individuals. In discussions with the less keen individual, the online facilitator helps him/her to identify how helping to reduce the partner’s stress is personally meaningful and relevant to their daily life together.

Throughout the program, online facilitators encouraged equal involvement between both couple members by incorporating aspects of both partners’ module feedback in crafting their own response to the couple. Such communication reflected engaging the couple individually and as a unit. For example, as illustrated in the following excerpt, the online facilitator highlights the individual and shared perspectives of a couple:

*It looks like you both got something out of the module and were able to really appreciate each other’s finer qualities, and the way in which you complement one another. I like* [the female participant’s] *comment about how the exercise was a “confidence builder,” and* [the male participant’s] *recognition of your “collective strengths.”*

At times, partners expressed having had a different experience of a particular module. In such cases, the online facilitator recognized the discrepancy, but also looked for and highlighted common ground in their responses. For instance, Module 4 is an exercise designed to assist the couple develop a sense that cancer is a common enemy by having the couple create a shared metaphor in relation to the illness using image, collage, or poetry (see [70]). In their feedback to this module, one couple indicated that they viewed their journey quite differently. The female participant described how she “was surprised to see [the image]. Very different. I have heard that art therapy helps people so it’s nice to see a more creative exercise” whereas her spouse commented that he found the exercise “...kind of boring, but [my partner] likes art so it was okay.” In providing feedback, the online facilitator validated both perspectives and at the same time drew their attention to the fact that they were able to work together despite it not being desirable to one of the partners:

*You two had quite a different reaction to this module exercise.* [Male Participant]*, despite finding this module “kind of boring,” you were able to join in with* [Female Participant] *and her enjoyment with this creative exercise, and come up with a shared concept together.*

### Challenge #2: Responding to Heightened Relationship Distress

Women with breast cancer and their partners, particularly younger couples, are understandably more distressed [62,66]. A couple can only cope as well as they have in the past, and coping with a diagnosis of cancer is especially complicated when there is preexisting relationship discord. The online facilitator’s challenge is how to simultaneously validate the couple’s feelings while containing their distress so that they may benefit from the program. In Module 1, which is intended to highlight a couple’s individual and collective strengths, one woman identified preexisting relationship difficulties and shared how she felt upset by the exercise as “it was very evident to me what our weaknesses are...it just highlighted for me how hurt I am and how hurt our relationship is.” Her male partner similarly voiced how he found the exercise difficult as it served to highlight the way in which “we need to work more on communication...I need to focus more attention on our relationship.” The online facilitator can incorporate a number of strategies in responding to such a situation, as demonstrated in the next few examples starting with the following that includes normalizing and empathetically responding to their distress:

It sounds like it was beneficial to a certain degree for the two of you to think about the positive qualities that you see in each other, as well as the strengths of your relationship, which can be especially difficult to do during stressful times. On the other side, sometimes sitting down to examine even positive aspects of the relationship can draw attention to the more difficult parts. It sounds like this happened to some degree for both of you.

By providing psychoeducation about the module and the program in general, the online facilitator tried to motivate the couple to persist and assure them that the program could provide them with an opportunity to work on their communication skills. The online facilitator also tried to unite the couple by drawing their awareness to the degree that they share in the concerns about their relationship:

Looking at your relationship a little more closely, and the way you interact, is a big part of this program—and you’ll see the exercises are designed to get both of you to do this. We believe that the first step to improving the relationship is being able to take a good look at it—what is working and what can be improved. The first few exercises focus more on building that relationship awareness so that you can communicate and problem-solve better in the long run. Nevertheless, I am glad to see that you were able to identify some meaningful attributes in each other and the relationship. It seems that both of you are aware that communication is an area in your relationship that needs attention. While enhancing communication is an implicit part of each module, there is one module in particular that explicitly focuses on this and provides specific guidance.

In addition to the preceding feedback, the online facilitator reiterated her availability to the couple, both online and by telephone. Although they did not seek out greater involvement, it is important for participants to know that this is an option. In addition, the online facilitator highlighted how she viewed the couple’s willingness to engage in this process, and look at their own behavior, as positive:

*Although it seems like this was a difficult exercise on a certain level, it is excellent that you,* [Male Participant]*, were able to identify what you could be working on as a couple, and also in terms of modifying your own behavior.*

### Challenge #3: Reinforcing Virtual Connection and Overcoming Silence

The convenience of an online program can also be a drawback as the lack of IP contact may engender less accountability. Some couples tend to delay completion of the weekly modules and fall out of touch. In order to keep such “straggler” couples engaged and avoid disconnecting from the program, the online facilitator reinforces structure and commitment to the program by communicating his or her own commitment, presence, and availability on a regular basis. The online facilitator’s responses are meant to encourage a couple’s progress while not seeming overly demanding. For instance, when a couple did not complete their module as scheduled, the online facilitator sent the following response:

*Hi* [Female Participant] *and* [Male Participant]*—I haven’t heard from you in a while so just wanted to check in and see how you are doing. Please touch base whenever you have a moment, even if it is just to let me know that you have been busy. I look forward to hearing back from you.*

The goal of such a communication is to express the way in which any response is preferable to no response and to open the door to communication.

The online facilitator will also send “gentle reminders” and assume that in the case of silence the couple may be having difficulty carving out some time in their schedule to do the exercise. At times, however, the couple may require more than a gentle reminder and when a couple has not responded to online communication, telephone contact initiated by the online facilitator is necessary. Similar to IP counseling, in connecting with the couple, the online facilitator communicates from a nonjudgmental, curious, and supportive stance. This means, for example, that she is open to hearing and accepting with respect to the couple’s reasons for delays, even if it is critical of some element of the program, and tries to best support the couple in order to help them overcome any obstacles that they are facing. As well, the online facilitator can review a couple’s progress within a module and send them a message validating the steps that they have completed and highlighting what remains to be done as a way of signaling her engagement with them.

### Challenge #4: Health Concerns and Changes in Health Status

Breast cancer tends to be more aggressive, more likely to recur, and more fatal in younger women [74-76]; hence, the possibility of a change in health status and ongoing worry with respect to health are very real concerns for this population. Thus, the online facilitator needs to be prepared to sensitively address such a situation by empathizing with the distress, giving the couple time to recover, and encouraging program continuance when the concerns have been resolved. This situation emerged for 2 couples during the pilot phase of the project. In one case, the male participant of one couple directly communicated his concerns to the facilitator:

*Sorry for the delay but we have had a bit of a fright.* [My wife] *found a lump on her neck which is a swollen lymph node and had an ultrasound, at which point the doctors decided that a biopsy is best. It looks suspicious. We are very worried as you can imagine and this is a priority right now...touch base with us in a week or two and we’ll let you know how things are going. I don’t think we will be doing the exercises until we know what the story is. I hope you understand.*

The online facilitator addressed this email immediately with a response that reflected support and concern for the couple:

Thanks for letting me know. I am very sorry to hear that. I will be thinking of the two of you and sending warm thoughts.

Given the magnitude of the concern, rather than following up with the couple through the Dialogue Room, the online facilitator called the couple a few weeks later to check in. Thankfully, the results of the female participant’s tests were clear. The couple expressed how stressed they had been and seemed grateful for the opportunity to discuss the stress they had undergone in the past few weeks. They also indicated that they were ready to resume with the program and the online facilitator sent them a message through the Dialogue Room letting them know that she set them up for the next module. This message allowed the online facilitator the opportunity to emphasize their we-ness in the very real and recent dealings with the fear of recurrence:

Whew! I am so relieved and happy to hear for the two of you that everything is okay, and the results were clear. I am sure that the two of you are very relieved. Doctor’s orders—please go out and celebrate! Feel free to start the next module at any time. I hope that this will be a good creative outlet for the two of you, and it will be interesting to see the way in which the two of you represent your shared experience especially given what you’ve just been through. Kindest regards and thinking of you both!

Another point to note here is the online facilitator’s appropriate use of humor within the framework of employing her skills as a clinician to support and empathize with the couple, while also keeping them on task with respect to their progression through the program. Also, she suggests that the next module may be a way for the couple to process their recent difficult experience in relation to cancer thereby demonstrating how preestablished exercises may be positioned to incorporate fluidly the couples’ fluctuating experiences and corresponding needs. Although health concerns may arise similarly in IP approaches, it is important to note that in an online intervention where there is no set appointment time and the online facilitator is remote, couples may find it easier to delay their involvement while facing a health crisis, making it important for the online facilitator to communicate her patience, empathy, and availability to the couple.

## Conclusion

To date, there has been very little discussion in the online intervention literature on the role of the online clinician, particularly using asynchronous interaction. An online facilitator in the context of online couple interventions is particularly important to manage engagement of both partners and maximize the couple’s learning. We propose that online therapeutic facilitation—a supporting role of the online clinician that (in the case of Couplelinks) focuses on encouraging the couple’s bond as well as adherence to the intervention—is a useful clinical construct to guide more effective online therapist involvement with couples. Therapeutic online facilitation of couples is distinct from IP couple interventions, the latter being more intense and often entails more liberal use of interpretation and inference. However, online facilitators have several other tools at their disposal that can enhance outcomes, such as being able to review progress made by the couple as well as being available for immediate feedback and support at any time throughout the week. The clinical perspective on online facilitation presented here was derived over the course of developing and applying an online intervention for couples affected by breast cancer. Research on whether adherence to such principles contributes to outcomes has yet to be evaluated. Nonetheless, the principles presented here may offer guideposts for clinicians in the process of developing an online support program entailing asynchronous interaction between client and facilitator, stimulate discussion with respect to the theoretically necessary components of online facilitation, and identify research questions for future studies.

## Abbreviations

IP: in-person
RCT: randomized controlled trial
